# CXCL12-CXCR4/CXCR7 Axis in Cancer: from Mechanisms to Clinical Applications

**DOI:** 10.7150/ijbs.82317

**Published:** 2023-06-26

**Authors:** Yaru Yang, Jiayan Li, Wangrui Lei, Haiying Wang, Yunfeng Ni, Yanqing Liu, Huanle Yan, Yifan Tian, Zheng Wang, Zhi Yang, Shulin Yang, Yang Yang, Qiang Wang

**Affiliations:** 1 Department of Orthopedics, Shenmu Hospital, Faculty of Life Sciences and Medicine, Northwest University, Shenmu, China.; 2 Key Laboratory of Resource Biology and Biotechnology in Western China, Ministry of Education. Faculty of Life Sciences and Medicine, Northwest University, Xi'an, China.; 3 Department of Thoracic Surgery, Tangdu Hospital, The Airforce Medical University, Xi'an, China.; 4 Department of Cardiothoracic Surgery, Central Theater Command General Hospital of Chinese People's Liberation Army, Wuhan, China.

**Keywords:** Chemokine CXCL12, CXCR4, CXCR7, Cancer progression, Inhibitors

## Abstract

Cancer is a multi-step disease caused by the accumulation of genetic mutations and/or epigenetic changes, and is the biggest challenge around the world. Cytokines, including chemokines, exhibit expression changes and disorders in all human cancers. These cytokine abnormalities can disrupt homeostasis and immune function, and make outstanding contributions to various stages of cancer development such as invasion, metastasis, and angiogenesis. Chemokines are a superfamily of small molecule chemoattractive cytokines that mediate a variety of cellular functions. Importantly, the interactions of chemokine members CXCL12 and its receptors CXCR4 and CXCR7 have a broad impact on tumor cell proliferation, survival, angiogenesis, metastasis, and tumor microenvironment, and thus participate in the onset and development of many cancers including leukemia, breast cancer, lung cancer, prostate cancer and multiple myeloma. Therefore, this review aims to summarize the latest research progress and future challenges regarding the role of CXCL12-CXCR4/CXCR7 signaling axis in cancer, and highlights the potential of CXCL12-CXCR4/CXCR7 as a biomarker or therapeutic target for cancer, providing essential strategies for the development of novel targeted cancer therapies.

## Introduction

Cancer is a leading cause of premature death worldwide 1. The emergence of cancer as a significant challenge facing humanity cannot be overstated 2. The development of cancer is attributed to the accumulation of genetic mutations and/or epigenetic alterations. Initially, normal cells undergo a transformation into a tumor state, acquiring the ability to proliferate uncontrollably, resist cell death, and induce angiogenesis. Subsequently, the ability to invade, disseminate, and survive at secondary sites leads to metastasis, which is the primary cause of death in most cancer patients 3. Furthermore, tumor recurrence and the lack of effective treatment options contribute to high mortality rates 4. Therefore, comprehending the mechanisms underlying tumorigenesis and progression is crucial for unraveling the intricate biology of cancer.

Chemokines are a class of small cytokines (about 8-17 kDa) that play a crucial role in regulating cell migration and localization during development, homeostasis, and inflammation. They are produced by various immune cells, endothelial cells, and mesenchymal cells, and bind to G protein-coupled receptors 5, 6. The chemotactic properties of chemokines enable them to guide immune effector cells to the site of inflammation and coordinate the interaction between immune cells 7. Currently, there are approximately 50 human chemokines and 20 receptors that have been identified 8. Based on their functions, chemokines can be classified two categories: inflammatory and homeostatic chemokines. The former is only induced by the innate immune system in response to infection and injury (i.e., pathogen-associated molecular patterns and damage associated molecular patterns), and can attract immune cells to the inflammatory site, thereby helping to amplify the immune response already underway, while the latter is produced constitutively. Chemokines can also be classified based on the position of the first two cysteine (C) residues in their protein sequences. There are four classes of chemokines: CC-chemokines, CXC-chemokines, C-chemokines, and CX3C-chemokines. CXC-chemokines are characterized by an additional amino acid between the first and second cysteines. Among them, CXCL12 is the most extensively studied member of the chemokine family. Historically, CXCL12 has been considered a homeostasis chemokine, but recent studies have also attributed some inflammatory activities to this chemokine 9. CXCL12 interacts with its specific receptors CXCR4 and CXCR7 to induce downstream signaling pathways, which have extensive effects on gene expression, cell chemotaxis, proliferation and migration. CXCL12 and its receptor signaling axis are involved in various diseases, including cancer, atherosclerosis 10, Alzheimer's disease 11, diabetes 12, and autosomal dominant polycystic kidney disease 13. Significantly, the CXCL12-CXCR4/CXCR7 signaling axis plays a central role in tumor cell proliferation, angiogenesis, invasion, tumor microenvironment, and chemotherapy-induced drug resistance.

In recent years, there has been a significant advancement in the understanding of the role of CXCL12 and its receptor in tumor progression. However, a comprehensive and systematic summary of this topic is still lacking. Therefore, this review sheds light on evaluating the role of the CXCL12-CXCR4/CXCR7 signaling axis in cancer. First, we briefly review the biological structure and activation pathways of CXCL12 and its receptors. Next, we describe the role of CXCL12 and its receptors in tumor progression. Subsequently, we outline the role of the CXCL12-CXCR4/CXCR7 signaling axis in the development of various cancers, including leukemia, breast cancer, lung cancer, prostate cancer and multiple myeloma. Finally, we summarize the current application of drugs based on targeting the CXCL12-CXCR4/CXCR7 signaling axis in tumor therapy and propose several potential new research directions for further studies. Overall, this review provides a comprehensive and systematic evaluation of the role of the CXCL12-CXCR4/CXCR7 signaling axis in cancer, which can contribute to the development of new therapeutic strategies for cancer treatment.

## Biological structure and activation pathway of CXCL12 and its receptors

### CXCL12

Chemokines can be subdivided into four categories according to the position of the first two cysteine residues in their protein sequences, including the CC-chemokines, the CXC-chemokines, C-chemokines, and CX3C-chemokines. CXCL12, originally known as stromal cell-derived factor-1 (SDF-1) or pre-B-cell growth-stimulating factor (PBSF), belongs to the CXC chemokine subfamily and is characterized by the first and second cysteines separated by another amino acid. CXCL12 participates in the homeostatic regulation of leukocyte trafficking and tissue regeneration 14, 15, and plays an important role in the homing of bone marrow (BM) stem cells 16, cardiovascular development 17, and neuronal guidance 18.

To date, seven CXCL12 subtypes (α, β, γ, δ, ε, θ, and the predicted isoform iso7) have been identified from selective gene splicing on chromosome 10. Among these subtypes, α and β, consisting of 89 and 93 amino acids respectively, have been the most studied 19. The most common α subtype is involved in various physiological or pathological processes, such as myoblasts migration during myogenesis and muscle regeneration 20, management of hematopoietic stem cell populations in the BM, and neuromodulation in the central nervous system (CNS) 21. The β subtype contains additional four amino acids, which is consistent with its stronger pro-angiogenic properties. It mainly exists in hypervascular organs such as kidneys, liver, and spleen 22. The γ subtype is highly expressed in organs with low vascularization, including heart and brain 23. The γ subtype does not possess as high a potency as the α or β isoforms, but has the highest affinity for the CXCR4 binding site and the longest duration of downstream effects 24. Especially, CXCL12-γ significantly increases the breast cancer metastatic tropism for BM 25. The functions of the three other identified isoforms of CXCL12 remain unclear.

### CXCL12-CXCR4 signal axis

**Figure 1:**
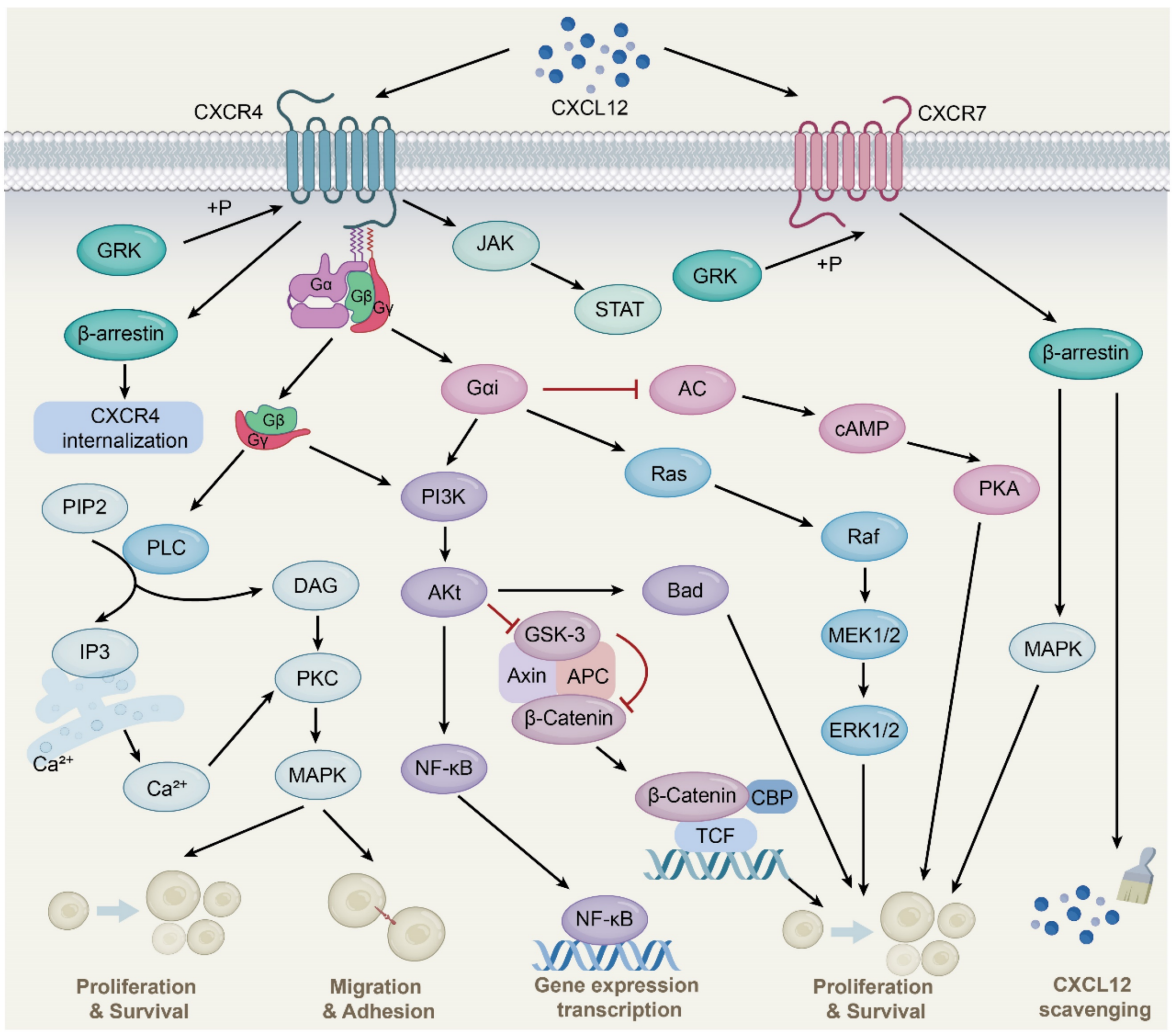
** CXCL12-CXCR4/CXCR7 signaling pathway.** CXCL12 is widely expressed in various tissues, such as liver, brain, lung, kidney, heart, spleen and bone marrow. Upon CXCL12 binding to CXCR4, the G protein is dissociated into Gα and Gβγ subunits, which interacts with their downstream target proteins and regulates downstream signaling pathways, respectively. The Gβγ subunit activates PLC, which results in the conversion of PIP2 to DAG and IP3 and the release of Ca^2+^, followed by PKC activation and phosphorylation of target proteins. Gαi and Gβγ subunits activate PI3K-AKt pathway. Subsequent AKt activation regulates gene expression via the NF-қB pathway. Activated AKt also resulted in GSK-3 inactivation and β-Catenin stabilization. Stable β-Catenin enters the nucleus and activates gene transcription, thereby promoting cell proliferation. AKt activation also affects cell proliferation and survival by phosphorylation of inactivated B-cell lymphoma-associated cell death agonists. In addition, the Gαi subunit induces Ras protein kinase activation, which is subsequently transduced by MAPK signaling, including phosphorylation of ERK1/2. The Gαi subunit also inhibits cAMP production, thereby activating downstream pathways. Moreover, the binding of CXCL12 to CXCR4 activates the JAK/STAT pathway independent of the G protein. CXCL12 also mediates CXCR4 receptor internalization through GRK-dependent phosphorylation and subsequent interaction of CXCR4 with β-arrestin. Upon CXCL12 binding to CXCR7, no classical GPCR-mediated signaling is observed, instead signaling preferentially through β-arrestin proteins.

CXCR4 is a 7-transmembrane G-protein coupled receptor (GPCR) that is highly expressed in many cell types such as lymphocytes, endothelial cells, hematopoietic stem cells, stromal fibroblasts, and cancer cells 26. The G protein is located on the inner side of the cell membrane and comprises three different subunits, namely α, β, and γ. Upon interaction with CXCL12, CXCR4 triggers the dissociation of the heterotrimeric G protein into Gα and Gβγ subunits and converts guanosine diphosphate bound to the G protein to guanosine triphosphate, followed by the activation of downstream signaling (Fig. 1) 27. These changes further activate Ras protein kinase 28, phosphatidylinositol-3-kinase (PI3K) 29, and phospholipase C (PLC) 30 signaling and release intracellular calcium ions, leading to gene transcription, intracellular calcium increase, and the occurrence of cell chemotaxis, survival, and proliferation. Additionally, the CXCL12-CXCR4 signaling axis can activate Janus kinase/signal transducer and activator of transcription (JAK/STAT) 31, Wnt/β-catenin 32, and Ca^2+^/calmodulin dependent protein kinase II/AMP-response element-binding protein (CaMKII/CREB) 33 signaling pathways.

### CXCL12-CXCR7 signal axis

CXCR7, also known as atypical chemokine receptor 3 (ACKR3), is a GPCR expressed in various cell types, including hematopoietic cells, neurons 34, and activated endothelial cells. Interestingly, unlike the typical chemokine receptor CXCR4, CXCR7 binding ligands is unable to activate the typical signaling pathway through the Gα subunit, but preferentially transmits signals through the non-classical β-arrestins pathway 35. As adaptor and signal transducer proteins, β-arrestins include arrestins 1, 2, 3, and 4, which were originally found to inhibit GPCR-mediated signaling, competing with G protein to bind GPCRs 36. Subsequent studies found that ligand binding CXCR7 activated GPCR kinase 2 and receptor phosphorylation through Gβγ subunit, followed by β-arrestin2 (an important member of the arrestin family) recruitment, thereby mediating downstream signaling 37. This is due to the lack of the DRY-LAIV motif in CXCR7; the DRY-LAIV motif is essential for Gαi protein coupling and calcium signaling pathways 38, 39. In contrast, the DRYLSIT motif was found in CXCR7, and this structural difference resulted in CXCR7's inability to signal through Gαi 40. In addition, recent studies have also shown that CXCR7 activates intracellular signaling pathways, including protein kinase B (AKT) and JAK/STAT 41, 42.

Importantly, CXCR7 has a much higher affinity for CXCL12 than CXCR4. CXCR7 reduces CXCL12 levels and attenuates CXCR4 activity, at least in part, by recruiting β-arrestin2, heterodimerizing with CXCR4, and scavenging CXCL12. CXCR7 is an important negative regulator of CXCL12 expression 43, 44. CXCR7 acts as a scavenger to internalize and deliver CXCL12 for lysosomal degradation, resulting in a gradient of CXCL12 in the extracellular environment to affect cell migration and other processes 45. This scavenging activity of CXCR7 is essential for maintaining the proper balance of CXCL12 levels in the extracellular environment. Therefore, CXCR7 plays a critical role in regulating CXCL12-mediated signaling pathways and cellular processes. Further studies are needed to fully understand the mechanisms underlying CXCR7-mediated regulation of CXCL12 and its potential as a therapeutic target.

It is worth noting that with the deepening of research, another ligand of CXCR7, interferon-inducible T-cell alpha chemoattractant (I-TAC)/CXCL11, has also been discovered. Similar to CXCL12, CXCL11 is a member of the chemokine family and is up-regulated in many tumors and controls tumor progression 46, 47. The discovery of CXCL11 adds complexity to the already intricate landscape of chemokine biology. Here, in order to clarify the role of CXCL12 and its receptor signal axis in tumors, the role of CXCL11 will not be discussed in this paper.

## The role of CXCL12-CXCR4/CXCR7 signaling axis in tumor progression

Chemokines and their receptors are overexpressed in various tumor cell types, including breast, prostate, lung, and pancreatic cancer cells, as well as tumor endothelial cells. Tumor-produced chemokines promote the recruitment of immunosuppressive cells to induce an immunosuppressive microenvironment 48. Moreover, the interaction between chemokines and receptors can promote the survival and proliferation of tumor cells, and promote neoangiogenesis of tumors 49. Of particular importance, there is substantial evidence that the CXCL12-CXCR4/CXCR7 signaling axis plays a central role in tumor cell proliferation, tumor metastasis, angiogenesis, and tumor immune escape.

### Tumor proliferation

Primary tumor growth is a complex multistage process involving in the regulation of multiple growth factors and signaling pathways 50. Both stromal- and tumor-derived CXCL12 can directly stimulate the growth and proliferation of tumor cells. In addition, CXCL12 inhibits the apoptosis of tumor cells, thereby supporting the survival of tumor cells.

The CXCL12/CXCR4/PI3K/AKT axis has emerged as a prognostic factor of adamantinomatous craniopharyngiomas (adaCP) progression and a potential biomarker for assessing recurrence tendencies. In detail, CXCL12-CXCR4 significantly increased the expression of β-catenin and the translocation of β-catenin to the nucleus through PI3K/AKT signaling pathway, and continuously promoted the transcription of target genes, thereby promoting the proliferation and migration of adaCP cells and the development of adaCP 51. CXCL12-CXCR4 also played an important role in breast cancer growth and metastasis by activating JAK2/STAT3 52. Importantly, the co-expression levels of CXCR4 and p-STAT3 in breast cancer tissues were correlated with tumor size, lymph node metastasis and histological grade of breast cancer 52. In addition, CXCR4 overexpression increased the expression of miR-15a/16-1, induced the down-regulation of B-cell chronic lymphocytic leukemia/lymphoma-2 (BCL-2) and Cyclin D1, and activated MAPK signaling pathways, thereby promoting neuroblastoma (NB) cell proliferation, NB growth and treatment resistance. These increased the difficulty of treatment 53. *In vitro* experiments on NB showed that after transfection of CXCR4-specific small interfering RNA (siRNA) into SH-SY5Y cells (human NB cells), the invasion capacity of SH-SY5Y cells was significantly reduced 54. During the progression of pancreatic intraepithelial neoplasia (PanIN) (the accepted precursor lesions to pancreatic duct cancer) in mice and humans, CXCL12-CXCR4 promoted precancerous lesions of pancreatic duct cancer by increasing the frequency and intensity of its expression, partially dependent on MAPK signaling. It is worth noting that there was a positive feedback loop that promoted CXCR4 expression and further amplified MAPK signaling, accelerating the proliferation of mouse PanIN cells and the progress of PanIN 55. In addition, the tumor biomarker circPVT1 promoted the proliferation of medullary thyroid cancer cells and activated CXCL12-CXCR4 signaling by targeting miR-455-5p 56. Transcription factor 12 (TCF12) could directly bind to the CXCR4 promoter to up-regulate the expression of CXCR4, thereby enhancing the proliferation, migration, and invasion of hepatocellular carcinoma cells. This may be related to the activation of MAPK/ERK and PI3K/AKT signaling pathways by CXCL12-CXCR4 57. Moreover, CXCL12 gene silencing significantly inhibited the proliferation and invasion of colon cancer DLD-1 cells by down-regulating the MAPK/PI3K/AP-1 signaling pathway 58. Leucine-rich repeats containing 4 (LRRC4), a putative glioma suppressive gene, significantly inhibited the proliferation, chemotaxis and invasion of human glioblastoma U251 cell line induced by CXCL12/CXCR4/ERK1/2/AKT 59.

Studies involving immunodeficient mice subcutaneously engrafted with A549 human lung tumor fragments have shown that the CXCR7 antagonist, CCX754, inhibited lung tumor growth 60. Furthermore, CXCR7 gene silencing inhibited the proliferation and invasion of colon cancer cells and induced the apoptosis of colorectal cancer (CRC) cells by decreasing the expression of p-ERK, proliferating cell nuclear antigen, matrix metallopeptidase (MMP)-2, while increasing the expression of caspase-3 61. In addition, on one hand, CXCR7 activated Src kinase phosphorylation in a β-arrestin2-dependent manner to enhance the proliferation of melanoma cells. On the other hand, it also regulated melanoma angiogenesis and vascular endothelial growth factor (VEGF) secretion to promote melanoma progression 62.

### Tumor metastasis

Metastasis is a complex and multifaceted process that is a major contributor to cancer-related mortality. It involves the sequential steps of tumor initiation at the primary site and subsequent spread to distant organs 63. First, the basement membrane ruptures, and tumor cells invade the nearby stroma. Subsequently, tumor cells access the circulation and infiltrate the parenchyma of the target organ. Finally, tumor cells adapt to the microenvironment and further proliferate to form large metastases 64.

In human CRC samples, CXCR4 and integrin αvβ6 (an important adhesion receptor on CRC cells) were to be overexpressed and significantly associated with liver metastasis. Further studies revealed that the CXCL12-CXCR4 signaling axis upregulated the expression of integrin αvβ6 by increasing ERK phosphorylation and subsequent Ets-1 transcription factor activation, leading to the directional transfer of human CRC cell line Caco-2 and the development of CRC 65. In addition, upregulation of homeobox B5 (HOXB5), a member of the HOX family, promoted CRC metastasis through transactivation of the metastasis-related gene CXCR4 and integrin subunit β3. Importantly, CXCL12 upregulated the expression of HOXB5 through the CXCR4-ERK1/2-Ets-1 pathway, which formed the CXC12-HOXB5-CXCR4 positive feedback loop and promoted the progression of CRC 66. Moreover, CXCL12 secreted by cancer associated fibroblasts (CAFs) reduced the tight junctions of endothelial cells and down-regulated the expression of adherence junction proteins, leading to increased vascular permeability and tumor cell infiltration into the lung 67. Matrix metalloproteinases (MMPs), proteases secreted by tumor cells, destroy the structure of extracellular matrix and the tumor microenvironment and promote cancer cell migration 68. In endometrial cancer, CAFs activated PI3K/AKT and MAPK/ERK signals in a paracrine-dependent manner, or increased MMP-2 and MMP-9 secretion in an autocrine-dependent manner through CXCL12-CXCR4 axis, thus promoting the invasion and metastasis of endometrial cancer 69. Importantly, a large number of studies have focused on targeted inhibition of the CXCL12-CXCR4 axis in breast cancer. Saikosaponin A, a triterpenoid glycoside, is the main active component of the plant Radix bupleuri and has anti-cancer activity 70. It possessed anti-tumor growth and metastasis inhibition effects through regulating the PI3K/AKT/mTOR and MMPs signaling pathways by reducing the expression of CXCR4 in triple-negative breast cancer (TNBC) cells 71. During epithelial-mesenchymal transition (EMT), cancer cells lose their cell-cell adhesion properties and acquire invasive and migratory properties. A dipeptidyl peptidase (DPP)-4 inhibitor accelerated breast cancer metastasis by inducing EMT through CXCL12/CXCR4/mTOR axis, while metformin countered the harmful effect of DPP-4 inhibitor on breast cancer metastasis 72.

Moreover, the CXCL12-CXCR7 axis has also been implicated in tumor metastasis. In mice of breast cancer induced by the 4T1.2 breast cancer cell line which has been shown to highly metastasize to the lung, the CXCL12-CXCR7 axis mediated metastasis of breast cancer cells by activating proinflammatory STAT3 signaling and angiogenic marker vascular cell-adhesion molecule-1, accelerating breast cancer progression. CXCL12-CXCR7 axis also recruited tumor-promoting macrophages to tumor sites by regulating the macrophage colony-stimulating factor/macrophage colony-stimulating factor receptor signaling pathway. And macrophage infiltration further enhanced breast tumorigenicity and invasiveness 73.

### Tumor angiogenesis

A tumor is a rapidly proliferating mass of cells, in which angiogenesis is an important rate-limiting step for tumor formation and progression 74.

CXCR4 was found to be significantly expressed in CD133^+^ glioma stem-like cells (GSCs), indicating that GSCs may have mediated tumor angiogenesis through CXCR4 75. Further studies have found that CXCL12-CXCR4 axis induced CD133^+^ GSCs to produce VEGF through PI3K/AKT signaling pathway, thereby promoting the growth and angiogenesis of GSCs-induced glioma 75. In addition, hypoxia-inducible factor-1 (HIF-1) is an upstream inducer of VEGF. CXCR4 was significantly upregulated in hypoxic pseudopalisading cells around the necrotic area with HIF-1α overexpression and induced the expression of VEGF through the nuclear transcription factor YinYang1, thereby promoting angiogenesis in tumor progression 76, 77. Furthermore, the tumor endothelial cells isolated from mouse super-metastatic malignant melanoma xenografts secreted CXCL12, and the binding of CXCL12 and CXCR7 promoted angiogenesis in the tumor microenvironment through the activation of ERK1/2 78. These findings suggest that CXCR7 may be a novel target for anti-angiogenic therapy.

### Immunosuppressive tumor microenvironment

The tumor microenvironment (TME) is the primary site of interaction between tumor cells and the host immune system, and its homeostasis is crucial for cancer progression 79, 80. Abnormal CXCL12 secretion promotes the differentiation and infiltration of immunosuppressive cells into tumors, including regulatory T cells (Treg cells) 81, tumor-associated macrophages (TAM) 82 and myeloid-derived suppressor cells (MDSC) 83. This process creates an immunosuppressive microenvironment that hinders the immune system's ability to recognize and eliminate cancer cells.

TAM are a significant marker of unfavorable prognosis in cancer, as they are associated with increased tumor progression, neoangiogenesis, and immune escape. Jose and his team established a melanoma model in mice with resolved sepsis and found that CXCL12-CXCR4 mediated increased TAM accumulation in sepsis-surviving mice. Further studies using AMD3100 (a CXCR4 antagonist) showed that post-sepsis and uninfected mice survived longer than control group mice 84. In addition, the high infiltration of Treg cells can also mediate the formation of an immunosuppressive environment. In nasopharyngeal carcinoma (NPC) cells, CXCL12 effectively attracted CXCR4^+^ Treg cells. Specifically, Epstein-Barr virus (EBV, an NPC infection-caused virus) mediated a feedback loop of TGF-β1-SMAD3-PI3K-AKT-c-JUN-miR-200a-CXCL12-CXCR4 through its nuclear antigen EBNA1 to regulate Treg cells infiltration 85. Nataša and group found that the tumor-associated inflammatory mediator prostaglandin E_2_ (PGE_2_) regulated CXCL12 production in malignant ascites of ovarian cancer patients, as well as CXCR4 expression on MDSC and its response to CXCL12. However, blocking of PGE_2_ eliminated the migration of MDSC to ascites microenvironment 86. Moreover, cytolytic T lymphocytes (CTL) can kill some antigenic substances such as tumor cells. In subdermal tumor xenograft mice, CXCL12 overexpression induced an immunosuppressive microenvironment and reduced CTL infiltration by recruiting immunosuppressive cells 48.

## The role of CXCL12-CXCR4/CXCR7 signaling axis in cancer

The CXCL12-CXCR4/CXCR7 signaling axis plays a central role through autocrine/paracrine mechanisms in the progression of various tumors, including leukemia, breast cancer, lung cancer, prostate cancer, and multiple myeloma.

### Leukemia

Leukemia is a type of hematological malignancy that is characterized by the presence of diffuse dysplasia of leukocytes in the BM 87. The interaction between leukemic cells and the BM matrix leads to the migration of leukemic cells into the BM microenvironment nest, thereby promoting the proliferation and migration of leukemic cells.

Lauren and colleagues investigated the role of the CXCL12-CXCR4 signaling axis in the progression of T cell acute lymphoblastic leukemia (T-ALL). They transplanted T-ALL cells from leukemic mice into the BM of healthy mice and observed that leukemic cells had a vascular niche and directly interacted with CXCL12-producing stromal cells. Furthermore, CXCL12-deleted mice reduced leukemic cells expansion and tumor burden, and no splenomegaly or thymic infiltration compared with the control group 88. These findings suggest that vascular endothelial cells accelerate leukemia progression through producing CXCL12. In addition, the downstream kinases Src and ABL1 were activated by CXCL12-CXCR4, leading to the phosphorylation of Rho GDP-dissociation inhibitor 2 (RhoGDI2) at Y153, Y24, and Y130. Phosphorylation of Y24 and Y153 resulted in the release of RhoA and RhoC from RhoGDI2. Activated RhoA and RhoC led to CXCR4-mediated migration of T-ALL cell lines to CXCL12, triggering leukemic tumor cells infiltration 89. Besides, CXCL12 promoted histone H3K9 methylation in primary T-ALL cells within minutes, which altered the global chromatin configuration and promoted the nuclear deformation and migration efficiency of T-ALL cells, further aggravating the tumor 90. In pediatric patients with B cell acute lymphoblastic leukemia (B-ALL), the infiltration of tumor cells in the liver or spleen was more serious in patients with high expression of CXCR4 91. In addition, CXCR4 acts as a marker of CNS infiltration in ALL. Specifically, zeta-chain-associated protein kinase 70 (ZAP70) up-regulated CXCR4 expression through ERK1/2 signaling and guided CXCR4 migration to ligand, thus resulting in more severe CNS involvement in T-ALL patients with high ZAP70 and CXCR4 expression 92. Importantly, the CXCL12-CXCR4 axis is a key driver of the chemotherapy resistance during leukemia treatment. Chemotherapy up-regulated CXCR4 expression in ALL cells. Co-culture of ALL cells with BM stromal cells (which can produce CXCL12) developed drug resistance, and protected ALL cells from chemotherapy-induced apoptosis, leading to further deterioration of leukemia 93. In conclusion, the CXCL12-CXCR4 signaling axis plays a key role in regulating the growth, migration, infiltration, and chemoresistance of leukemia.

Furthermore, the CXCL12-CXCR7 signaling axis plays a crucial role in leukemia by regulating migration, metastasis, and homing of leukemia cells. CXCR7 is highly expressed in ALL cells compared with myeloid or normal hematopoietic cells. Silencing CXCR7 by lentivirus targeting in leukemia cells significantly slowed their migration 94. Tissue factor pathway inhibitor (TFPI) improved CXCL12-mediated migration of chronic lymphocytic leukemia (CLL) cells by increasing CXCR7 expression, suggesting that CXCL12-CXCR7 was involved in organ infiltration in CLL patients 95. MicroRNA-101 (miR-101) is a novel inhibitor of T-ALL that directly targets CXCR7. T-ALL xenograft or metastasis mice were established by the subcutaneous or intravenous injection of leukemia cells stably expressing a miR-101 precursor or shCXCR7. Compared with the control groups, the tumor growth and lung metastasis were significantly slowed down in the miR-101 overexpression group or CXCR7 knockdown group. Furthermore, miR-101 inhibited T-ALL tumor development by targeting CXCLI2/CXCR7/STAT3 signaling pathway activation 96. In addition to migration, CXCR7 also promoted CXCL12-mediated homing of leukemic and normal hematopoietic cells (U937 and CD34 cells) to the BM and spleen of mice 97. In conclusion, studies targeting the biological functions of the CXCL12-CXCR7 signaling axis may provide better therapeutic interventions for leukemia patients.

### Breast cancer

Breast cancer is a malignant tumor formed on the mammary epithelial tissue. The progression of this disease is often attributed to the metastasis of tumor cells. The CXCL12-CXCR4/CXCR7 signal axis has been identified as a crucial factor in the malignant transformation and migration these cells.

The POU1F1 transcription factor, also known as Pit-1, has been found to be expressed in the mammary gland, and its overexpression promoted tumor growth and metastasis 98. In a pro-tumor model induced by Pit-1, Pit-1 exerted a pro-angiogenic effect through increasing the expression of CXCR4 and CXCL12. Furthermore, high levels of CXCL12 in metastatic target tissues (such as liver and lung) acted as chemoattractant for primary tumor cells with high CXCR4 expression, and CXCR4 blockade significantly reduced metastasis to these tissues 99. In metastatic breast cancer (mBC) mice, the CXCR4 antagonist Plerixafor significantly reduced fibrosis, increased CTL infiltration, and improved immune suppression by inhibiting CXCR4 signal transduction. And finally, the metastatic rate of mBC decreased 100. Hence, CXCL12 and CXCR4 can be considered as key therapeutic targets for mBC to reduce the metastasis rate of cancer.

There are still some controversies on the role of CXCR7 in breast cancer. High levels of CXCR7 were present in the cancer tissues of breast cancer patients. In mice with primary and metastatic breast cancer, CXCR7 overexpression significantly promoted breast tumor growth and enhanced experimental lung metastasis in immunodeficient mice 101. CXCR7 overexpression also enhanced primary tumor growth and angiogenesis in severe combined immunodeficient mice (which have a higher incidence of tumors). Notably, the invasion and metastasis of cancer cells in mice overexpressing both CXCR4 and CXCR7 were significantly reduced, possibly due to the distinct roles of CXCR4 and CXCR7 in metastasis. Specifically, CXCR4 mainly mediated breast cancer invasion, while CXCR7 inhibited invasion but promoted primary breast tumor growth through angiogenesis 102. In addition, CXCR7 on the surface of breast cancer cells reduced CXCR4 signaling by internalizing and degrading CXCL12. In adult mice with CXCR7 specifically knocked out in the vascular endothelium, implantation of AT-3 breast cancer cells into mice significantly increased the local recurrence of cancer and the number of circulating tumor cells 103. This result suggests that endothelial CXCR7 limits breast cancer metastasis in the metastatic cascade and provides a new direction for drug development targeting CXCR7 in cancer.

### Lung cancer

Metastasis is a major contributor to the poor prognosis and high recurrence rate of lung cancer. EMT is a key pathological mechanism in the early metastasis of primary lung cancer. The abnormality of CXCL12-CXCR4/CXCR7 signaling axis plays a crucial role in EMT, invasion and chemotherapeutic drug resistance of tumor cells.

CD133 is a common cancer stem cell marker. In non-small cell lung cancer (NSCLC) A549 cell line, CD133^+^CXCR4^+^ cells had a stronger migration ability, higher expression of Vimentin (one of the mesenchymal phenotypes) and lower expression of E-cadherin (one of the epithelial phenotypes) than CD133^+^CXCR4^-^ 104. These results indicate that CXCR4 is involved in CD133-induced migration and EMT in NSCLC. Additionally, CAFs isolated from lung adenocarcinoma tissue produced CXCL12 in a conditioned medium, and CAF-derived CXCL12 enhanced EMT and cancer aggressiveness by upregulating the expression of CXCR4, β-catenin, and peroxisome proliferator-activated receptor δ 105. Similarly, CXCR7 overexpression promoted primary tumor growth and metastasis in A549 cells 106. However, CXCR7 silencing significantly inhibited TGFβ1-induced migration, invasion, and EMT of cancer cells *in vitro*, and reduced tumor sphere-forming capacity, stem-like properties, and chemoresistance 107. Notably, drug resistance is a key reason for the poor prognosis of lung cancer. In A549 lung cancer cells, CXCL12-CXCR4 promoted anti-apoptotic activity by activating JAK2/STAT3 pathway, greatly reducing the efficacy of chemotherapeutic drugs (such as cisplatin) 108. In addition, the EMT phenotype of NSCLC cells show *de novo* resistance to epidermal growth factor receptor tyrosine kinase inhibitors (EGFR TKI, tumor therapeutic drugs). Becker and colleagues found that CXCR7 silencing not only re-sensitized EGFR TKI in NSCLC with a mesenchymal phenotype and EGFR mutation, but also promoted partial mesenchymal transition to the epithelium 109. This result suggests that co-targeting EGFR and CXCR7 may provide new ideas for the treatment of EGFR-TKI-resistant NSCLC patients by promoting mesenchymal transformation to epithelial cells.

### Prostate cancer

Prostate cancer is a malignant tumor occurring in the prostate epithelium 110. Bone metastasis is the leading cause of mortality in patients with prostate cancer. The current studies on CXCL12-CXCR4 may provide potential signal transduction pathways and targets for the clinical treatment of prostate cancer.

Extensive research has demonstrated that the CXCL12-CXCR4 axis is one of the key factors of bone metastasis in prostate cancer. Activation of this pathway increased EMT markers (E-cadherin and Vimentin) in prostate cancer cells, leading to the loss of adhesion and the acquisition of invasion and migration ability to promote prostate cancer. However, knockdown of CXCR4 significantly reduced the migration and invasion of prostate cancer cells into osteoblasts, thereby improving bone metastasis 111. Besides, the CXCL12γ isoform has been shown to promote cancer development in castration-resistant prostate cancer (CRPC). On the one hand, it induced cancer stem cells phenotype via CXCR4-mediated PKCα/NF-қB activation, ultimately increasing the number of disseminated tumor cells in the soft and bone tissues. On the other hand, CXCL12γ induced an aggressive neuroendocrine phenotype in prostate cancer cells that was commonly seen in patients with recurrent prostate cancer, and promoted growth, metastasis, and chemical resistance of metastatic CRPC 112. In addition, CXCL12-CXCR4 is a marker of bone metastasis in prostate cancer and affects prostate cancer angiogenesis through tumor endothelial cells. It is uncovered that CXCL12 has potential to be a novel endothelial cell marker for prostate-specific tumors. Its expression was significantly up-regulated in tumor endothelial cells compared with endothelial cells* in vitro*. In a mouse model of prostate cancer xenograft, CXCL12-CXCR4 inhibition exerted the ability of anti-angiogenic through reducing the number and density of blood vessels 113. Decreased CXCL12 expression inhibited the expression of MMP9 mediated by zinc-finger transcription factors and reduced the metastasis of prostate cancer cells 114.

### Multiple myeloma

Multiple myeloma (MM) is a heterogeneous plasma cell neoplasm which is characterized by clonal proliferation and accumulation of malignant plasma cells and produces myeloma proteins in BM or extra-medullary tissues, thereby leading to lytic bone degeneration, osteopenia, renal diseases, hypercalcemia and anemia 115. The CXCL12-CXCR4 axis is closely related to growth, metastasis and drug resistance of MM. Therefore, understanding the mechanisms underlying this axis is essential for effective therapeutic strategies for MM.

MM cells increased connexin 43 expression in mesenchymal stromal cells, leading to an increase in CXCL12 levels and stimulating CXCR4 expression on MM cells. This enhances mitochondrial transfer from mesenchymal stromal cells to MM cells, promoting MM growth by supporting oxidative phosphorylation and increasing ATP production 116. Yazan and colleagues injected MM cells treated with AMD3100 (a CXCR4 inhibitor) into mice, and found that MM cells continued to circulate in peripheral blood in CXCR4-inhibited mice, while MM cells in control mice rapidly exited the blood circulation and entered the BM 117. This study suggests that the CXCL12-CXCR4 axis promotes migration of MM cells to BM. Interestingly, enhanced CXCL12-CXCR4 signaling in the BM was found to restrict MM cells from leaving the primary BM and re-entering the blood circulation, so increased CXCR4 expression in BM MM plasma cells led to reduced tumor dissemination and better prognosis 118-120. Besides, CXCL12γ enhanced the adhesion ability of BM stromal cells and MM cells *in vitro*, resulting in drug resistance caused by cell-cell interaction mediated by adhesion molecules 121. Furthermore, CXCL12 was found to increase the expression of survivin, BCL2 and the multidrug resistance transporter ABCC1 in MM cells to enhance anti-apoptotic and detoxification ability, therefore promoting MM cells survival 122. In conclusion, these finding provides a better strategy for the treatment and prognosis of MM patients in the future.

## Research on targeting CXCL12-CXCR4/CXCR7 signal axis in tumor therapy

**Figure 2:**
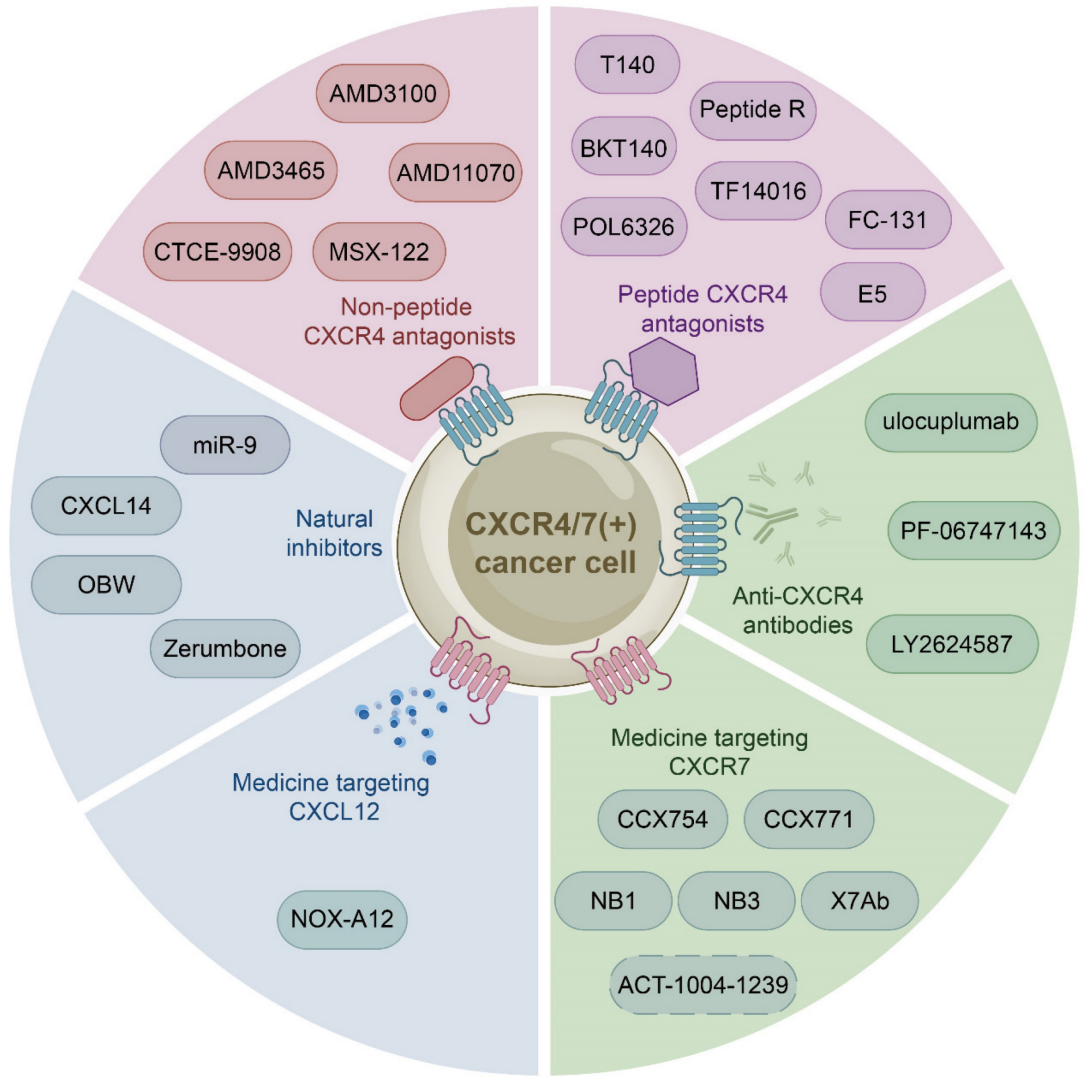
** Medicine targeting the CXCL12-CXCR4/CXCR7 signaling axis.** Medicine targeting the CXCL12-CXCR4/CXCR7 signaling axis can classified into six categories: non-peptide CXCR4 antagonists, peptide CXCR4 antagonists, anti-CXCR4 antibodies, medicine targeting CXCR7, medicine targeting CXCL12, and natural inhibitors.

The growing body of investigations have reported that the CXCL12-CXCR4/CXCR7 signaling axis plays a crucial role in a variety of cancers, including leukemia, breast cancer, lung cancer, prostate cancer, MM, colorectal cancer, and bladder cancer. Therefore, the identification of CXCL12-CXCR4/CXCR7 signaling axis antagonists is a promising target for tumor therapy. So far, several tumor therapeutic drugs targeting this axis have been reported (Fig. 2). They can inhibit the growth of cancer cells and exhibit promising anticancer activity in a variety of tumor cells (Table 1).

### Medicine targeting CXCR4

Initially developed for the treatment of human immunodeficiency virus, CXCR4 inhibitors have gained attention for their potential use in the treatment of hematological and solid malignancies. With further study of the CXCL12-CXCR4 axis, a growing number of medicine targeting CXCR4 have been discovered. These drugs can be classified into three categories: non-peptide CXCR4 antagonists such as AMD3100, peptide CXCR4 antagonists such as BTK140, and CXCR4 antibodies such as ulocuplumab.

#### Non-peptide CXCR4 antagonists

Non-peptide CXCR4 antagonists, including AMD3100, AMD3465 123, 124, AMD11070 125, 126, MSX-122 127, and CTCE-9908 128, have been identified as potential therapeutic for cancer treatment. And one of them AMD3100 (commercially known as Plerixafor, a bicycline), the only Food and Drug Administration (FDA) approved specific small molecule antagonist targeting CXCR4, prevents CXCR4 from binding to CXCL12 to inhibit calcium mobilization, chemotaxis, and GTP binding 129, 130. Specifically, AMD3100 blocks the binding of one glutamine and two aspartic acid residues in CXCR4, while inhibiting the conformational changes required for intracellular kinases activation 131, 132. However, many anticancer drugs, such as AMD3100, are ineffective due to tumor resistance. Therefore, extensive investigations have focused on the combination of AMD3100 and other anticancer drugs to obtain better cancer treatment outcomes currently. For example, combretastatin A4 nanodrug (CA4-NPs) is a vascular disrupting agent, which can selectively destroy immature tumor vessels and inhibit tumor growth 133. AMD3100 and CA4-NPs combination significantly inhibited tumor growth and metastasis in breast cancer mice 134. Additionally, a pre-clinical study proposed to combine AMD3100 with bortezomib to overcome drug resistance in relapsed/refractory MM. The mechanism of action involved inhibiting CXCR4, which made MM cells less adherent and more sensitive to bortezomib treatment. Subsequent clinical phase I/II trials showed that the combination therapy was well tolerated and had a high response rate in patients received bortezomib previously 135, 136. Besides, Xue and colleagues proposed a new targeting delivery system for the treatment of ovarian cancer by modifying paclitaxel-loaded PEGylation bovine serum albumin nanoparticles with AMD3100. This drug delivery system both targeted tumors and improved therapeutic efficacy with a high safety profile* in vitro* and *in vivo* 137. Moreover, a phase I study has shown that combining AMD3100, sorafenib (an FLT3-ITD inhibitor), and G-CSF (clettable CXCL12) resulted in an increased response rate to treatment in patients with relapsed/refractory FLT3-ITD mutant acute myelogenous leukemia (AML) 138. Together, AMD3100 has been shown to be more potent in combination with a variety of anticancer drugs than alone, so further studies in larger trials are warranted to confirm these results and apply them to the clinic.

#### Peptide CXCR4 antagonists

Peptide CXCR4 antagonists include T140, BKT140, peptide R, T22, POL6326 139, TF14016 140, and FC-131 141. Among these synthetic peptides, T140 is considered to be the most active CXCR4 peptide antagonist. Preclinical studies have demonstrated the efficacy of T140 and its analogues in blocking CXCR4 in various cancers, including breast cancer, melanoma and leukemia 142, 143. BKT140 (4F-benzoyl-TN14003), a T140 analogue, is a novel selective inhibitor of CXCR4. Compared with AMD3100, it has higher affinity and stronger binding force. BKT140 inhibited apoptosis of AML cells through the AKT/ERK pathway* in vitro* and *in vivo* 144. BKT140 had better safety and efficacy in the phase I clinical study of MM patients, and significantly increased the mobilization of CD34^+^ cells and lymphocytes to promote the recovery of immune function in MM patients 145. Bockorny and lab found that BKT140 and pembrolizumab (a humanized monoclonal anti-PD1 antibody) combination led to partial tumor regression by reducing the accumulation of immunosuppressive cells, thus enhancing objective response rates and disease control rates of chemotherapy in pancreatic cancer comparing with other therapies 146. Another phase II study showed that the combination of BKT140 and cytarabine was safe in the treatment of relapsed/refractory AML. BKT140 made cytarabine treatment more effective by mobilizing AML cells and transferring them to peripheral blood related to clinical response, and ultimately prolonged the survival time of AML patients 147.

A new CXCR4 antagonist peptide R was developed in 2013 148. Peptide R enhanced the efficacy of anti-programmed cell death 1 (PD-1) in mouse models of colon cancer and melanoma 149. The combination treatment of peptide R and anti-PD-1 reduced the infiltration of forkhead box P3 (FoxP3) positive cells (a major marker of Treg cells), tumor growth, anti-PD-1 sensitivity and tumor drug resistance 149. In 2016, Caterina and colleagues developed a peptide R-modified stealth liposome (PL-Peptide R) with better efficacy and biodistribution compared to peptide R alone. PL-Peptide R inhibited CXCR4-dependent migration* in vitro*, and reduced lung metastasis and increased survival in melanoma mice *in vivo* 150. Furthermore, PL-peptide R-DOX (the PL-peptide R loaded with doxorubicin) treatment reduced the effective dose (the concentration of the drug required to halve the number of viable cells after treatment) of DOX IC50 and had less lung metastasis compared to treatment with PL-DOX in experimental mice 150. The combination of PL-peptide R and DOX has better anti-tumor metastatic effects and may be a promising therapeutic strategy for the treatment of metastatic tumors.

The novel peptide E5 has been shown to be more stable and simpler to prepare than BKT140. E5 blocked CXCL12-mediated endothelial progenitor cell recruitment and slowed tumor angiogenesis by inhibiting AKT and ERK pathways 151. Moreover, E5 improved the efficacy of multiple chemotherapy drugs by enhancing the sensitivity of breast cancer cells to chemotherapy drugs such as paclitaxel, elemene and cisplatin 151. To increase the dissolving stability of E5 in physiological buffer solutions and provide an E5 delivery platform, Jie and colleagues developed E5 micellar preparation (M-E5), which significantly prolonged the survival of refractory AML mice 152. In summary, E5 is expected to be a candidate targeted drug for the treatment of breast cancer and refractory AML. Further research is necessary to validate its effectiveness and safety through more extensive clinical trials.

#### CXCR4 antibodies

CXCR4 monoclonal antibodies, including ulocuplumab, PF-06747143, LY2624587, are mainly used in the treatment of hematological malignancies.

Ulocuplumab (BMS-936564/MDX1338) is a human immunoglobulin G4 (IgG4) that prevents interaction between CXCR4 and CXCL12 through binding to the extracellular second loop of CXCR4, thereby inhibiting CXCL12-mediated signaling and cell migration 153. Ulocuplumab reduced the migration and invasion of RH30 alveolar rhabdomyosarcoma cell lines* in vitro*. Furthermore, ulocuplumab combined with activated and expanded natural killer cells (which are highly cytotoxic to RH30 cells) *in vivo*, showing that ulocuplumab effectively inhibited invasion and metastasis of rhabdomyosarcoma cells 154. A phase I clinical study of Waldenström macroglobulinemia conducted by Steven and colleagues demonstrated that the combination of ibrutin and ulocuplumab resulted in a shorter primary response time, attainment of major responses, and longer 2-year progression-free survival of patients 155. Besides, a phase Ib/II trial demonstrated a favorable safety profile and good tolerability of ulocuplumab in combination with lenalidomide and dexamethasone in relapsed/refractory myeloma patients, and the combined treatment also had high response rates in patients previously treated with immunomodulatory agents 156.

PF-06747143 is a novel IgG1 that exhibits high affinity binding to CXCR4, thereby preventing CXCL12-induced calcium flux and cell migration. This antibody has demonstrated strong cytotoxicity and high therapeutic value for CLL cells 157. Importantly, PF-06747143 has excellent efficacy in combination with a variety of standard of care agents in hematologic tumors, such as bortezomib in MM mice and daunorubicin plus cytarabine in patient-derived xenograft mice resistant to AML chemotherapy 158.

LY2624587, a fully humanized CXCR4 monoclonal antibody, can effectively block the binding of CXCL12 and CXCR4, induce dose-dependent cell death in human hematological cancer cells *in vitro* and *in vivo*, and is used for the treatment of hematological malignancy patients 159, 160.

### Medicine targeting CXCR7

There are few therapies targeting CXCR7 in preclinical models. Medicine targeting CXCR7 such as small molecules CCX266, CCX662, CCX733, CCX771, and X7Ab, have been developed to inhibit β-arrestin signaling, thereby inhibiting proliferation, growth, and metastasis of tumor cells. Treatment with CCX754 or CCX771 compounds reduced tumor growth in the lungs of mice inoculated with colon cancer cells. Notably, the CXCR7 antagonist did not affect the extent of liver metastasis in colon cancer mice, suggesting that CXCR7 may mediate cancer cell metastasis in an organ-specific manner 161. In addition, llama-derived immunoglobulin single variable domains (Nanobodies) such as NB1 and NB3 have been identified to specifically target CXCR7. These nanobodies inhibited the binding of CXCL12 and blocked the recruitment of β-arrestin2 to CXCR7, thereby reducing the secretion of angiogenic factors in head and neck cancer cell lines 162. Furthermore, a single chain anti-CXCR7 antibody (X7Ab) has been developed to bind to the same receptor site as CXCL12, inhibiting CXCL12-mediated receptors activation. X7Ab has the same Fc sequence as human immunoglobulin G1, and participates in anti-tumor immune response of glioblastoma cells through Fc-driven antibody-dependent cytotoxicity and cellular phagocytosis. Moreover, the combination of X7Ab and temozolomide enhanced the activation of M1 macrophages to support anti-tumor immune response and prolonged the survival time of glioblastoma mice 163. Moreover, ACT-1004-1239 is a recently discovered potent, insurmountable (meaning reduces the maximum effect of agonists), oral CXCR7 antagonist with good drug tolerance and safety 164, 165. ACT-1004-1239 has been shown to be effective in acute lung injury and inflammatory demyelinating diseases such as multiple sclerosis in preclinical animal models 166, 167. However, whether ACT-1004-1239 has a protective effect in cancer remains to be further explored.

### Medicine targeting CXCL12

NOX-A12 is a pegylated mirror oligonucleotide that specifically binds to CXCL12 with high affinity, thereby inhibiting the binding of CXCL12 to CXCR4/CXCR7. In hematological tumors, NOX-A12 mobilized leukocytes, hematopoietic stem cells and progenitor cells into the peripheral blood, thereby promoting chemotherapy drugs to kill tumor cells 168. Notably, NOX-A12 not only directly bound and antagonized CXCL12, but also indirectly neutralized CXCL12 by competing with glycosaminoglycans such as heparin. These resulted in the release and neutralization of CXCL12 from glycosaminoglycans bound to the surface of stromal cells and reduced CXCL12 levels in BM and other tissues 169. Besides, Meggy and colleagues revealed high safety and good drug tolerance of NOX-A12 treatment in patients with advanced metastatic colorectal and pancreatic cancer 170. Additionally, Liu and team demonstrated that the combination of radiotherapy and NOX-A12 not only slowed down tumor growth, but also prolonged the survival time of rats with N-ethyl-N-nitrosourea-induced brain tumors 171. These findings suggest that NOX-A12 may have potential as an agent for the treatment of various cancers.

### Natural Inhibitors

In addition to synthetic medicines, natural inhibitors that interfere with the CXCL12-CXCR4/CXCR7 axis in different ways are also being developed. MiR-9 has been reported to inhibit the Wnt/β-catenin signal pathway by slowing the expression of CXCR4, thereby reducing the proliferation of oral squamous cell carcinoma 172. CXCL14, a natural inhibitor of the CXCL12-CXCR4 axis, specifically bound to CXCR4 with high affinity and inhibited CXCL12-mediated chemotaxis and migration of human bone marrow-derived hematopoietic progenitor cells and leukemia-derived cells 173. Zerumbone, a component of subtropical ginger (*Zingiber zerumbet*), is a novel CXCR4 expression inhibitor that reduced CXCR4 expression in breast cancer by down-regulating NF-κB signaling. It further inhibited ligand-induced invasion of breast cancer and pancreatic cancer 174. Furthermore, onbaekwon (OBW), a complex herbal formulation, inhibited the expression of CXCR4 in colon cancer cells, breast cancer cells and other cells in a concentration- and time-dependent manner by blocking the endogenous activation of NF-κB, and abolished CXCL12-induced invasion of colon cancer cells 175.

## Conclusions and future directions

CXCL12 interacts with CXCR4 and CXCR7, and stimulates several downstream signaling, which have a wide range of effects on cell chemotaxis, proliferation and migration. Much effort has been done on the roles of CXCL12-CXCR4/CXCR7 axis in various stages of cancer. This review aims to provide an overview of the progress made in understanding the signaling axis of CXCL12 and its receptors in various types of cancer.

Tumor development is a complex process involving multiple factors. The change of internal environment leads to abnormal expression of related pathway factors, and balance of immune system is broken. The expression levels of CXCL12 and its receptors in cancer tissues differ significantly from those in normal tissues. In most cases, the CXCL12-CXCR4/CXCR7 signaling axis promotes tumor cell proliferation, metastasis, angiogenesis and the formation of an immunosuppressive tumor microenvironment. Importantly, abnormalities of this signaling axis contribute to cancer progression, leading to unfavorable disease outcomes and poor patient survival. Targeted therapy has become a hot topic in cancer treatment research due to its unique high efficiency and precision. Therefore, the screening of CXCL12-CXCR4/CXCR7 axis antagonists is expected to bring hope for cancer treatment. At present, many drugs targeting CXCR4 have been reported. Most of the drugs targeting CXCR7 remain in the preclinical stage and few drugs targeting CXCL12 have been found. Nevertheless, currently only AMD3100 has been approved for clinical treatment, and most of the other drugs are still some distance away from entering clinical translation (Table 2).

Importantly, the CXCL12-CXCR4/CXCR7 axis has emerged as a promising target for cancer immunotherapy. Immune checkpoint blockade (ICB) therapy improves the host immune system's attack on tumor cells by inhibiting the binding of programmed death receptors and their ligands 176. ICB therapy has played a role in the treatment of many types of tumors. For example, S100 calcium-binding protein A9 (S100A9)-CXCL12 promoted the expansion and accumulation of myeloid-derived suppressor cells in breast cancer, forming a microenvironment that allowed tumor growth to render breast cancer insensitive to ICB. The combination of S100A9-CXCL12 signaling axis inhibitors and αPD-1 antibody significantly inhibited growth of breast cancer cells 177. In addition, drugs targeting the CXCL12-CXCR4/CXCR7 signaling axis can also be combined with various drugs to achieve superior efficacy. However, the complex pathogenesis of tumors and the intricate association between CXCL12-CXCR4/CXCR7 signaling and other signaling pathways make it challenging to fully comprehend the role of CXCL12-CXCR4/CXCR7 in cancer. Besides, CXCL12-CXCR4/CXCR7 signaling is more complex than the CXCL12/CXCR4 axis, and due to the extremely complex chemokine-chemokine receptor network in the body, more components need to be considered when designing CXCR7 antagonists.

Other chemokine family members are also involved in the development of tumors. CCL2 participates in a variety of malignant tumors, such as breast cancer 178, lung cancer 179, and cervical cancer 180. CXCL8 and its receptors are overexpressed in various tumors and correlated with tumor stage and grade 181. However, it is important to note that chemokines have dual functions during tumor development 182. For instance, in addition to the CXCL12-CXCR4/CXCR7 signal axis, CXCR4 and CXCR7 significantly reduced the invasion and metastasis in breast cancer mice. This may be because CXCR4 mainly mediated breast cancer invasion, while CXCR7 inhibited invasion but promoted primary breast tumor growth through angiogenesis 102. Furthermore, chemokine system is involved in both recruitment of pro-tumor and anti-tumor immune cells. CXCR3 and its ligands CXCL9 and CXCL10, as well as CCR5 and its ligand CCL5, recruit both Treg cells (pro-tumor immune cell) and CTLs (anti-tumor immune cell) 183. Moreover, the chemokine system is complex and confounding. For example, CXCR4 binds only CXCL12, whereas CCR1 can bind to six different chemokine ligands. Therefore, inhibition of chemokine signaling may have broad systemic effects, resulting in adverse side effects 184.

The CXCL12-CXCR4/CXCR7 signaling axis has been identified as a critical player in cancer progression, and several novel drugs and combination therapy strategies targeting this pathway have been proposed. However, most of these approaches have only been validated in animal experimental models and have not yet been tested in clinical trials, so the clinical safety and efficacy still need to be further determined. In addition, the crosstalk between the CXCL12-CXCR4/CXCR7 signaling axis and other signaling pathways further complicates targeted therapy. This review comprehensively summarizes CXCL12-CXCR4/CXCR7 signaling axis in cancer progression and provides theoretical basis for the potential of CXCL12 and its receptors CXCR4/CXCR7 as cancer therapeutic targets.

## Figures and Tables

**Table 1:** Pre-clinical studies of CXCL12-CXCR4/CXCR7 signaling axis inhibitors/antagonists in tumors

Inhibitor/ Antagonist	Target	Cancer	Model	Effects	References
AMD3100 (+CA4-NPs)	CXCR4	Breast cancer	Mouse model of metastatic orthotopic 4T1 breast cancer	Tumor growth and metastasis were significantly reduced.	134
A delivery system by modifying paclitaxel-loaded PEGylation bovine serum albumin nanoparticles with AMD3100	CXCR4	Ovarian cancer	Xenograft mouse model of ovarian cancer cell line Caov3 cells	The number of metastatic lesions in mice was reduced.	137
AMD3465 (+D1MT)	CXCR4	Breast cancer	Mouse model of metastatic 4T1 breast cancer	It slowed bone metastasis and significantly prolonged survival in breast cancer mice.	123
AMD3465	CXCR4	Bladder cancer	Bladder cancer cell lines SW780	It inhibited the proliferation and migration of bladder cancer cells through CXCL12/CXCR4/β-Catenin axis.	124
AMD11070	CXCR4	Melanoma	Melanoma cell lines CHL-1 and A375	It inhibited melanoma cell migration.	125
AMD11070	CXCR4	Oral cancer	Oral cancer cell inoculation induced mouse model	It inhibited lung metastasis of oral cancer cells in mice.	126
MSX-122	CXCR4	Breast cancer, squamous cell carcinoma of the head and neck, and uveal melanoma	MDA-MB-231 breast cancer cell induced breast cancer mice, 686LN Ms cell injection induced cervical cancer mice, overexpression of HGF/TGF- β / CXCR4/MMP2 oelanoma OMM2.3 cells induced uveal melanoma micrometastasis mice	MSX-122 blocked cancer metastasis in mice.	127
CTCE-9908	CXCR4	Prostate cancer	Xenograft mouse model of prostate cancer cells	CTCE-9908 resulted in a decrement of tumor weight by about 20%.	128
BKT140	CXCR4	AML	Blasts of AML	It mediated AML cells apoptosis through Akt / ERK pathway.	144
Peptide R (+ anti-PD-1)	CXCR4	Colon cancer and melanoma	MC38 colon cancer and B16 melanoma-human CXCR4 transduction homologous mouse models	Tumor growth were reduced.	149
PL-peptide R	CXCR4	Melanoma	Melanoma B16-CXCR4 C57BL / 6 mice	It reduced lung metastasis and increased survival in melanoma mice.	150
Synthetic peptide E5	CXCR4	Breast cancer	Mouse model of breast cancer inoculated subcutaneously with 4T1 cells	It enhanced the efficacy of a variety of chemotherapy agents	151
M-E5	CXCR4	AML	Splenocyte of primary leukemia induced AML mouse model	M-E5 significantly prolonged the survival time of AML mice.	152
TF14016	CXCR4	Small cell lung cancer	Severe combined immunodeficiency mice inoculated with small cell lung cancer cells	It significantly inhibited the size and number of lung metastases.	140
Ulocuplumab	CXCR4	Rhabdomyosarcoma	RH30 alveolar rhabdomyosarcoma cell line	It reduced the migration and invasion of RH30 alveolar rhabdomyosarcoma cell line.	154
Ulocuplumab (+activated and expanded natural killer cells)	CXCR4	Rhabdomyosarcoma	Immunodeficient NSG mouse model implanted with CXCR4 RH30 cells	This therapy inhibited the invasion and metastasis of rhabdomyosarcoma cells.	154
PF-06747143 (+bortezomib)	CXCR4	MM	Mouse model of disseminated MM	The combination therapy reduced the burden of bone marrow tumors.	158
LY2624587	CXCR4	Hematological malignancies	Mouse xenograft models developed from human leukemia and lymphoma cells expressing high levels of CXCR4	It induced dose-dependent cell death in human hematological cancer cells.	159
CCX754 or CCX771	CXCR7	Colorectal cancer	Human HT29 colorectal cancer cells inoculation induced mouse model	Treatment with CCX754 or CCX771 reduced tumor growth.	161
Nanobodies (such as NB1 and NB3)	CXCR7	Head and Neck Cancer	Head and neck cancer cells (22A)	It reduced the secretion of angiogenic factors in head and neck cancer cell lines	162
X7Ab (+temozolomide)	CXCR7	Glioblastoma	Orthotopic xenograft of human glioblastoma in mice with severe combined immunodeficiency	Combination therapy prolonged the survival time of glioblastoma mice	163
NOX-A12 (+radiotherapy)	CXCL12	Brain tumor	N-ethyl-N-nitrosourea induced rat model of brain tumor	Combined treatment slowed down tumor growth and prolonged the survival time of model animals	171
MiR-9	CXCR4	Oral squamous cell carcinoma	Oral squamous cell carcinoma cell lines	MiR-9 reduced the oral squamous cell carcinoma proliferation.	172
CXCL14	CXCR4	—	THP-1, Jurkat (human T cell leukemia derived cell line), and BaF/3 (mouse pre-B cell line) cells	It inhibited CXCL12-mediated chemotaxis and migration of human bone marrow-derived hematopoietic progenitor cells and leukemia-derived cells.	173
Zerumbone	CXCR4	Breast cancer and pancreatic cancer	Breast cancer cell lines and pancreatic cancer AsPC-1 cells	It reduced CXCR4 expression in breast cancer and inhibited invasion of breast cancer and pancreatic cancer.	174
OBW	CXCR4	Colon cancer	The immortalized human colon cancer HCT116	OBW abolished CXCL12-induced invasion of colon cancer cells	175

**Table 2:** Clinical application & clinical trials of CXCL12-CXCR4/CXCR7 signaling axis inhibitors/antagonists in tumors

Inhibitor/ Antagonist	Company	Target	Cancer	Clinical Trial Number (status)	Trial Phase	Other indications
Clinical application:						
AMD3100	Sanofi Aventis	CXCR4	—	—	—	MM or non-Hodgkin lymphoma
Clinical trials:						
AMD3100 (+bortezomib)	Sanofi Aventis	CXCR4	MM	NCT00903968 (completed)	Phase 1/2	MM or non-Hodgkin lymphoma
AMD3100 (+sorafenib and G-CSF)	Sanofi Aventis	CXCR4	Relapsed/refractory FLT3-ITD mutant AML	NCT00943943 (completed)	Phase 1	
BKT140	BioLineRx	CXCR4	MM	NCT01010880 (completed)	Phase 1	Non-small cell lung cancer, non-hodgkin lymphoma, and AML
BKT140 (+pembrolizumab and chemotherapy)	BioLineRx	CXCR4	Pancreatic ductal adenocarcinoma	NCT02826486 (completed)	Phase 2	
BKT140 (+cytarabine)	BioLineRx	CXCR4	Relapsed/refractory AML	NCT01838395 (completed)	Phase 2	
POL6326 (+ eribulin)	Polyphor	CXCR4	HER2-negative metastatic breast cancer	NCT01837095 (completed)	Phase 1	Leukemia and MM
Ulocuplumab (+ibrutinib)	Bristol-Myers Squibb	CXCR4	Waldenström macroglobulinemia	NCT03225716 (active, not recruiting)	Phase 1/2	Hematologic malignancies
Ulocuplumab (+lenalidomide and dexamethasone)	Bristol-Myers Squibb	CXCR4	Relapsed/refractory MM	NCT01359657 (completed)	Phase 1	
NOX-A12	NOXXON Pharma	CXCL12	Advanced metastatic colorectal and pancreatic cancer	NCT03168139 (completed)	Phase 1/2	Chronic lymphocytic leukemia, and brain tumors

## Abbreviations

C: cysteine
SDF-1: stromal cell-derived factor-1
PBSF: pre-B-cell growth-stimulating factor
BM: bone marrow
CNS: central nervous system
GRK: GPCR kinase
PLC: phospholipase C
PIP2: phosphatidylinosital biphosphate
IP3: inositol triphosphate
DAG: diacylglycerol
PKC: protein kinase C
MAPK: mitogen-acticated protein kinase
GPCR: G-protein coupled receptor
PI3K: phosphatidylinositol3-kinase
AKT: protein kinase B
NF-қB: nuclear factor-қB
GSK-3: glycogen synthase kinase 3
Bad: B-cell lymphoma-associated cell death agonist
MEK1/2: mitogen-activated protein kinase kinase 1/2
ERK1/2: extracellular signal-regulated kinase1/2
AC: adenylyl cyclase
cAMP: 3': 5'-cyclic adenosine monophosphate
PKA: protein kinase A
JAK: Janus kinase
STAT: signal transducer and activator of transcription
CaMKII/CREB: Ca2+/calmodulin dependent protein kinase II/AMP-response element-binding protein
ACKR3: atypical chemokine receptor 3
I-TAC: interferon-inducible T-cell alpha chemoattractant
adaCP: adamantinomatous craniopharyngiomas
BCL-2: B-cell chronic lymphocytic leukemia/lymphoma-2
NB: neuroblastoma
siRNA: small interfering RNA
PanIN: pancreatic intraepithelial neoplasia
TCF12: Transcription factor 12
LRRC4: leucine-rich repeats containing 4
CRC: colorectal cancer
MMPs: matrix metallopeptidases
VEGF: vascular endothelial growth factor
HOXB5: homeobox B5
CAFs: cancer associated fibroblasts
TNBC: triple-negative breast cancer
EMT: epithelial-mesenchymal transition
DPP: dipeptidyl peptidase
GSCs: glioma stem-like cells
HIF-1: hypoxia-inducible factor-1
TME: tumor microenvironment
Treg cells: regulatory T cells
TAM: tumor-associated macrophages
MDSC: myeloid-derived suppressor cells
NPC: nasopharyngeal carcinoma
EBV: Epstein-Barr virus
PGE2: prostaglandin E2
CTL: cytolytic T lymphocytes
T-ALL: T cell acute lymphoblastic leukemia
RhoGDI2: Rho GDP-dissociation inhibitor 2
B-ALL: B cell acute lymphoblastic leukemia
ZAP70: zeta-chain-associated protein kinase 70
TFPI: Tissue factor pathway inhibitor
CLL: chronic lymphocytic leukemia
miR-101: microRNA-101
mBC: metastatic breast cancer
NSCLC: non-small cell lung cancer
EGFR TKI: epidermal growth
CRPC: castration-resistant prostate cancer
MM: multiple myeloma
FDA: Food and Drug Administration
CA4-NPs: combretastatin A4 nanodrug
AML: acute myelogenous leukemia
PD-1: programmed cell death 1
FoxP3: forkhead box P3
IgG4: immunoglobulin G4
OBW: onbaekwon
ICB: immune checkpoint blockade
S100A9: S100 calcium-binding protein A9
